# Source Causal Connectivity Noninvasively Predicting Surgical Outcomes of Drug‐Refractory Epilepsy

**DOI:** 10.1111/cns.70196

**Published:** 2025-01-03

**Authors:** Wentao Lin, Danni Yang, Chen Chen, Yuanfeng Zhou, Wei Chen, Yalin Wang

**Affiliations:** ^1^ School of Information Science and Technology Fudan University Shanghai China; ^2^ School of Materials Science and Engineering Lanzhou University of Technology Lanzhou China; ^3^ Human Phenome Institute Fudan University Shanghai China; ^4^ Children's Hospital of Fudan University Shanghai China; ^5^ School of Biomedical Engineering University of Sydney Camperdown New South Wales Australia; ^6^ School of Information Science and Engineering Lanzhou University Lanzhou China; ^7^ Key Laboratory of Special Functional Materials and Structural Design, Ministry of Education Lanzhou University Lanzhou China

**Keywords:** causal connectivity, drug‐refractory epilepsy, EEG source imaging, surgical outcome prediction

## Abstract

**Aims:**

Drug‐refractory epilepsy (DRE) refers to the failure of controlling seizures with adequate trials of two tolerated and appropriately chosen anti‐seizure medications (ASMs). For patients with DRE, surgical intervention becomes the most effective and viable treatment, but its success rate is unsatisfactory at only approximately 50%. Predicting surgical outcomes in advance can provide additional guidance to clinicians. Despite the high accuracy of invasive methods, they inevitably carry the risk of post‐operative infection and complications. Herein, to noninvasively predict surgical outcomes of DRE, we propose the “source causal connectivity” framework.

**Methods:**

In this framework, sLORETA, an EEG source imaging technique, was first used to inversely reconstruct intracranial neuronal electrical activity. Then, full convergent cross mapping (FCCM), a robust causal measure was introduced to calculate the causal connectivity between remodeled neuronal signals within epileptogenic zones (EZs). After that, statistical tests were performed to find out if there was a significant difference between the successful and failed surgical groups. Finally, a model for surgical outcome prediction was developed by combining causal network features with machine learning.

**Results:**

A total of 39 seizures with 205 ictal EEG segments were included in this prospective study. Experimental results exhibit that source causal connectivity in α‐frequency band (8~13 Hz) gains the most significant differences between the surgical success and failure groups, with a *p*‐value of 5.00e‐05 and Cohen's *d* effect size of 0.68. All machine learning models can achieve an average accuracy of higher than 85%. Among them, the SVM classifier with Gaussian kernel function and Bayesian optimization demonstrates the highest accuracy of 90.73%, with a PPV of 87.91%, an NPV of 92.98%, a sensitivity of 90.91%, a specificity of 90.60%, and an F1‐score of 89.39%.

**Conclusion:**

Our results demonstrate that the source causal network of EZ is a reliable biomarker for predicting DRE surgical outcomes. The findings promote noninvasive precision medicine for DRE.

## Graphical abstract

Herein, we propose the “source causal connectivity” framework for noninvasively predicting surgical outcomes of DRE. It integrates EEG source imaging, causal brain network analysis, statistical analysis, and machine learning classification. The SVM classifier with Gaussian kernel function and Bayesian optimization demonstrates the highest accuracy of 90.73%.

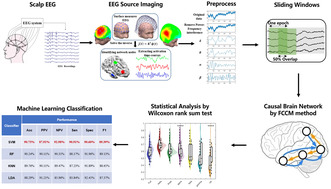

## 1 Introduction

Epilepsy, characterized by recurrent and unprovoked seizures, is a neurological disorder affecting tens of millions of individuals worldwide. Despite advancements in antiepileptic medications, a significant proportion of patients continue to experience seizures that are refractory to pharmacological interventions, called drug‐refractory epilepsy (DRE) [1]. For these patients, surgical intervention has been the most effective way to achieve seizure control and improve the overall quality of life. Epilepsy surgery involves the identification and resection or ablation of epileptic brain tissue, with the goal of eliminating or reducing the frequency and severity of seizures. Successful surgery requires complete removal or disconnection of the epileptogenic zone (EZ), whereas current surgical success rates are limited and unstable, varying from 40% to 80% [2]. Surgical failure can lead to potential consequences such as permanent nerve damage, and costly surgery is also a heavy financial burden. Accurate preoperative assessment of prognosis can help clinical experts adjust treatment plans in time. Therefore, exploring biomarkers for surgical outcomes and predicting surgical outcomes before neurosurgery has attracted considerable attention among clinicians [3, 4, 5, 6].

Traditional methods for predicting surgical outcomes include time‐domain, frequency‐domain, and time‐frequency domain analyses, each offering valuable information about EZ and seizure dynamics. For example, time‐domain analysis examines the temporal characteristics of EEG signals, such as epileptic spikes [7] and high frequency oscillations [5, 8]; frequency‐domain analysis focuses on the power spectrum of different frequency bands, and time‐frequency domain analysis, through Short‐time Fourier Transform (STFT), Wavelet Transform, etc., combines both aspects to provide a comprehensive view of the EEG signal characteristics during seizures. Network analysis methods have been increasingly applied to understand the complex interactions within brain regions [9, 10, 11, 12, 13, 14, 15, 16]. Instead of simply relating brain function to specific regions, such approaches focus on the collaborative functioning of the brain as a large‐scale network. In recent years, brain network analysis has shown remarkable success in predicting surgical outcomes for patients with DRE [17, 18, 19]. These predictive models leverage the rich information extracted from brain connectivity patterns to assess the likelihood of successful surgical intervention. Currently, the most widely used neurophysiological signal is invasive, that is, intracranial EEG (iEEG) recording [6, 7, 17, 20, 21, 22]. Though high‐quality signals from brain regions can be easily and directly obtained through iEEG, the complication risk is consequently increasing because of its invasiveness [23]. Moreover, iEEG has limited spatial resolution because its contacts record activity only in their direct vicinity and are blind to other brain regions. Thus, the actual focus may be missed leading to surgical failure [24]. Although invasive neurosurgery is necessary, the ability to predict surgical outcomes noninvasively in advance through scalp EEG would mitigate the risks associated with electrode implantation and reduce the substantial consequences of surgical failure. Therefore, noninvasive DRE precision medicine holds promising prospects.

Noninvasive neuroimaging, such as EEG and MEG, offers high temporal resolution and whole‐brain spatial coverage [25, 26, 27]. Very little work has been done on mapping noninvasively causal networks within EZs for patients with DRE. Noninvasive prediction of surgical outcomes has gradually attracted neuroscientists. However, poor prediction performance hardly promotes the clinical precision medicine [24]. A reliable noninvasive biomarker for predicting DRE's surgical outcomes is urgently needed. Causal brain network, closely related to the trigger mechanism and propagation drive of epileptic seizures, has shown superior performance in surgical outcomes invasive prediction with an accuracy of 92% [28]. However, there is little study on causal brain network in the noninvasive source space.

Reconstructing intracranial neural activity via multi‐channel scalp EEG, together with precise anatomical locations, is required. In this study, we employed EEG source imaging technology, that is, standardized low resolution brain electromagnetic tomography (sLORETA), to inversely remodel intracranial neuronal electrical activity with high spatial resolution through scalp EEG recordings [29]. After that, source signals from various brain regions can be obtained noninvasively, with which we performed causal brain network analysis within EZs. EZ is characterized by high‐frequency discharges and abnormal synchronization, which are closely related to the onset and propagation of seizures. Compared to non‐epileptogenic zones (NEZ), the networks within EZs show a higher correlation with epilepsy. Surgical outcomes depend heavily on whether the EZ is resected or disconnected, making the study of causal information flow within EZs even more valuable. This framework is novel and has potential in noninvasively predicting surgical outcomes for patients with DRE. Compared with previous studies, the contributions of this paper are:

1. We introduced a systematic technical framework, source causal connectivity, and noninvasively characterized the causal brain networks in the clinically‐annotated EZs for patients with DRE. The results demonstrate its feasibility and potential in noninvasive precision medicine for patients with DRE.
2. Based on the theory of attractor reconstruction, we developed full convergent cross mapping (FCCM), the most robust causal brain network measure, to characterize multi‐frequency epileptogenic brain network. Statistical analysis consistently shows a significant difference between surgical success and failure groups.
3. Combining the epileptic brain network features with machine learning algorithms, we completed the prediction of the surgical outcomes with satisfying performances at 90.73% for average accuracy.

## 2 Methods

### 2.1 Participants

The dataset used for this study was obtained from the Department of Neurology at the Children's Hospital of Fudan University and included 39 seizures from 10 patients aged 3–15 years. The diagnosis adhered to the criteria set by the International League against Epilepsy (ILAE). Patients with cancer, severe heart, lung, liver or kidney dysfunction, seizures triggered by neurological infections, poisoning, autoimmune disorders, those ineligible for surgical treatment, and those unable to be followed up after surgery for a variety of reasons were excluded from the study. Detailed participant information is listed in Table 1. Based on the Engel classification system widely used in clinical practice, the surgical outcomes are categorized into four classes: Class I (seizure‐free), Class II (rare seizures), Class III (worthwhile improvement), and Class IV (no worthwhile improvement). Among these patients, those classified as E I are considered as surgical success group, while those classified as E II~IV are together grouped as surgical failure group. The parents or legal guardians of each patient were given informed consent for this study. Data collection adhered to the clinical research regulations of the hospital and has been approved by the Ethics Committee of Children's Hospital of Fudan University (approval number: 2020‐521).

**TABLE 1:** Patient demographics and clinical information.

ID	Gender	Age (years)	MRI pathology	Follow up /year	Engel class	Clinical complexity	#Seizure	#Segment	Therapy	Surgical region
S001	M	13	N	3	E I	CC2	3	32	Resection	RT
S002	M	11	N	3	E IV	CC2	3	31	Resection	LMT
S003	F	7	P	3	E III	CC1	5	19	Resection	LI
S004	F	5.5	P	3	E I	CC1	4	7	Resection	LMF, LSF, LMFG, LSG
S005	M	8	P	3	E I	CC1	3	7	Resection	LMF, LSF
S006	F	6	P	2	E III	CC1	5	36	Resection	RI
S007	M	7	P	2	E I	CC1	5	10	Resection	LO
S008	M	4	P	2	E II	CC1	5	28	Resection	RT
S009	M	3	P	3	E I	CC1	3	17	Ablation	RT
S010	M	7	P	4	E I	CC1	3	18	Ablation	RF

*Note:* Gender: F, female; M, male; MRI Pathology: N, negative, non‐lesional patient; P, positive, lesional patient. Engel class: I, seizure free; II, significant seizure frequency reduction; III, slight seizure frequency reduction; IV, no change after surgery. Clinical complexity CC1~CC4 is ordered by increasing localization difficulty: lesional (CC1), focal temporal (CC2), focal extratemporal (CC3) and multifocal (CC4), CC2~CC4 are non‐lesional patients. Surgical region, (i.e., clinically annotated EZ): LI, left insula; LMF, left medial frontal lobe; LMFG, left middle frontal gyrus; LMT, left medial temporal gyrus; LO, left occipital lobe; LSF, left superior frontal gyrus; LSG, left straight gyrus; RF, right frontal lobe; RI, right insula; RT, right temporal lobe.

### 2.2 EEG Recordings and Preprocessing

**FIGURE 1:**
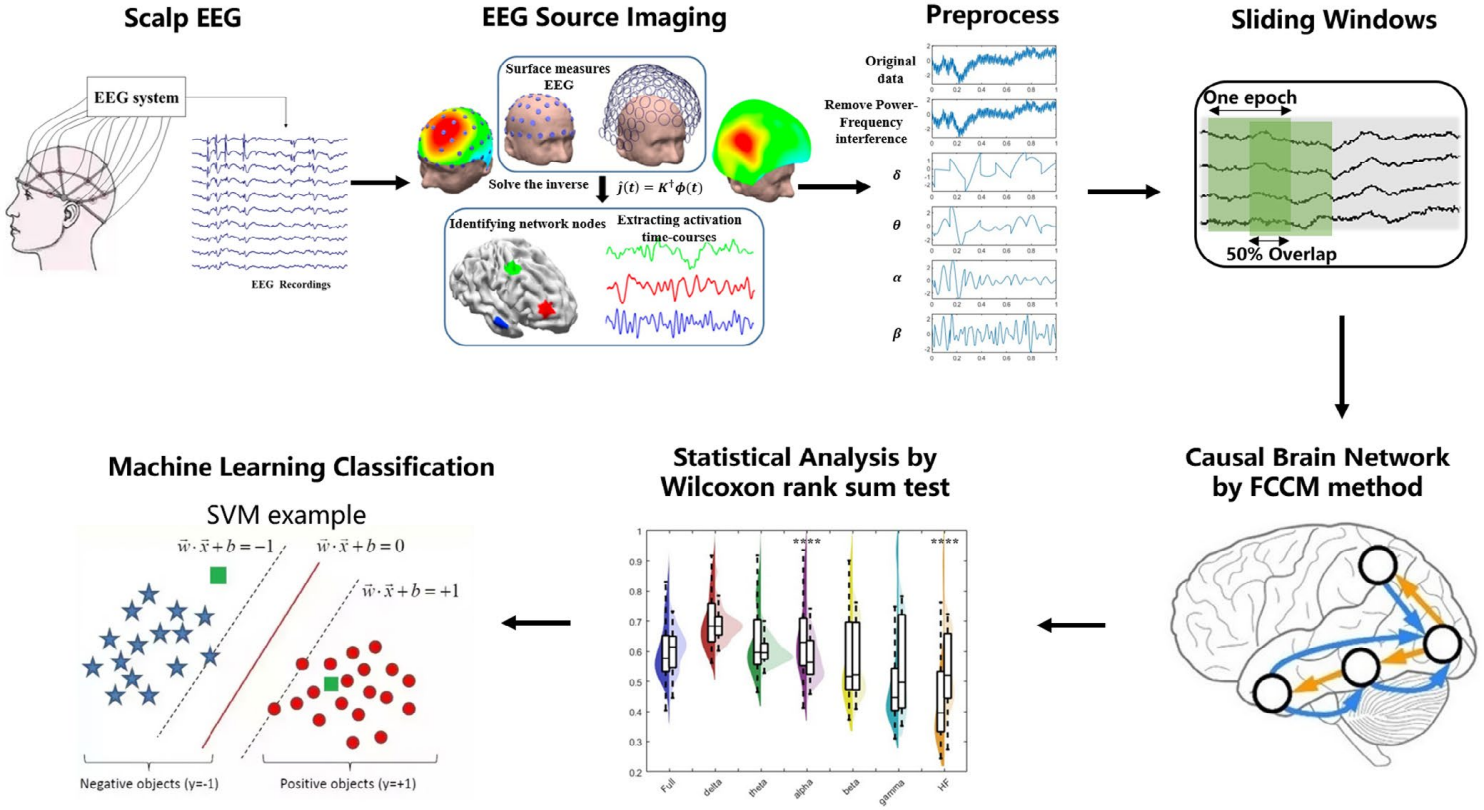
The flowchart of source causal connectivity.

The clinical continuous EEG recordings utilized in this study were obtained from Nihon Kohden EEG‐1200C based on the international 10–20 system. The recordings had a sampling rate of 500 Hz. In this research, EEG preprocessing was accomplished using EEGLAB and Brainstorm toolbox [30, 31] in MATLAB (R2022b, MathWorks Inc., USA). The preprocessing steps include: (1) Using a fourth‐order Butterworth filter to bandpass filter the EEG data between 0.5 and 300 Hz; (2) Notch filtering the data at 50 Hz and its harmonics with a stopband of 2 Hz to eliminate power frequency interference; (3) Discarding the “bad channels” deemed by the clinicians as broken or excessively noisy or artifactual from the dataset; (4) Perform Independent Component Analysis (ICA) using the EEGLAB toolbox to remove artifacts such as eye movements and muscle activity from the EEG signals. (5) Bandpass filtering the EEG data, remaining the frequency band of δ (0.5~4 Hz), θ (4~8 Hz), α (8~13 Hz), β (13~30 Hz), γ (30~80 Hz), and high frequency oscillations (HF, > 80 Hz). Note that the full frequency band EEG data without bandpass filtering was also included in the following analyzing. An overview of the proposed framework, source causal connectivity, is shown in Figure 1. The EEG Source Imaging part of the figure is adapted from [32]. In this section, we outline the methodology used for EEG source imaging, causal brain network analysis, statistical analysis, and machine learning classification.

### 2.3 EEG Source Imaging

High‐density EEG offers superior spatial resolution and more precise source localization compared to conventional EEG configurations. Nonetheless, the international 10–20 system remains the standard monitoring configuration employed in clinical practice. To ensure that our study is aligned with real‐world clinical scenarios and addresses practical concerns, we utilized data obtained from the international 10–20 EEG system. This choice facilitates the analysis and interpretation of results that are directly applicable to established clinical practices.

The scalp EEG recordings represent the linear summation of collective source activity [27, 29, 33]. Electrical potentials will attenuate as it propagates from intracranial neurons to the scalp, which is called volume conduction effects [34]. Reconstructing the intracranial nerve activity along with precise anatomical location through multi‐channel scalp EEG is especially needed. Current distributed source estimation methods include minimum norm estimation (MNE), dynamic statistical parametric mapping (dSPM), standardized low resolution electromagnetic tomography (sLORETA), exact low resolution electromagnetic tomography (eLORETA), etc. John et al. [35] have reported that sLORETA performed overall well compared with other methods with one of the highest AUC and lowest localization error for all SNR. Therefore, for our current experiment, standardized low resolution brain electromagnetic tomography (sLORETA) was applied to the EEG signals [36, 37]. In this measure, the scalp electrical potentials are modeled as:

(1) Φ=KJ+c1

where $\Phi \in {\mathbb{R}}^{N\times 1}$ is a vector containing scalp electric potentials measured at N cephalic electrodes. $K\in {\mathbb{R}}^{N\times \left(3M\right)}$ refers to the matrix of lead field, with $k_{i,l}=\left(k_{i,l}^{x},k_{i,l}^{y},k_{i,l}^{z}\right)$ is the scalp electric potential at the ith electrode due to a unit strength x‐, y‐, z‐ oriented dipole at the lth voxel. $J\in {\mathbb{R}}^{\left(3M\right)\times 1}$ is the matrix of the primary current density. c is an arbitrary constant allowing the use of any reference for the lead field and the measurements; and $1\in {\mathbb{R}}^{N\times 1}$ is a vector of ones. For EEG source localization, the aim is to estimate J, the primary current density. The function of interest is

(2) $$\begin{array}{c} F={\left|\left|\Phi -\mathrm{KJ}-c1\right|\right|}^{2}+\alpha {\left|\left|J\right|\right|}^{2}\; \end{array}$$

where α≥0 is a regularization parameter. This function is to be minimized with respect to J and c, for given K, Φ, and α.

(3) $$\begin{array}{c} \hat{J}=\min_{J,c}F=\min_{J,c}\;\left({\left|\left|\Phi -\mathrm{KJ}-c1\right|\right|}^{2}+\alpha {\left|\left|J\right|\right|}^{2}\right) \end{array}$$

Finally, $\hat{J}$ should be further scaled to become the source estimation of sLORETA. The specific solving process is omitted in this paper. For further details, please refer to [36]. The process of sLORETA Source Analyzing was carried out using Boundary Element Method (BEM) model of default anatomy and corrected through brainstorm toolbox of MATLAB to avoid affecting phase‐related information and causing problem to further causality measure. The results of EEG source analysis were presented in 74 x 2 (both left and right hemisphere) = 148 channels, based on the brain regions defined by the Destrieux Atlas. Subsequently, we regarded the clinically annotated surgical area in Table 1 as the EZ regions and manually labeled the channels corresponding to the EZ regions. For each patient, we used all the EZ channels to calculate the causal coupling strength, with the number of EZ channels ranging from 2 to 39.

### 2.4 Causal Brain Network Analysis

Inspired by recent study about convergent cross‐mapping (CCM), we proposed a newly methodology, FCCM, to perform causal brain network analysis. Considering a dynamics system with time series $X=x\left(t\right),Y=y\left(t\right),t\in \left[1,L\right]$, to estimate the causality X→Y, the attractor manifold $M_{X}$ of variate X is generated with embedding dimensionality m and time delay δ:

(4) $$\begin{array}{c} M_{X}={\left[X_{1}^{m,\delta },\ldots ,X_{i}^{m,\delta },\ldots ,X_{L-\left(m-1\right)\delta }^{m,\delta }\right]}^{T} \end{array}$$

where $\begin{array}{c} X_{i}^{m,\delta }=\left\{x\left(i\right),x\left(i+\delta \right),\ldots ,x\left(i+\left(m-1\right)\delta \right)\right\} \end{array}$.

Another attractor manifold $M_{Y}$ of series $Y=y\left(t\right)$ can also be obtained:

(5) $$\begin{array}{c} M_{Y}={\left[Y_{1}^{m,\delta },\ldots ,Y_{i}^{m,\delta },\ldots ,Y_{L-\left(m-1\right)\delta }^{m,\delta }\right]}^{T} \end{array}$$

where $\begin{array}{c} Y_{i}^{m,\delta }=\left\{y\left(i\right),y\left(i+\delta \right),\ldots ,y\left(i+\left(m-1\right)\delta \right)\right\} \end{array}$.

In (4)~(5), Takens' embedding theorem is utilized to build dynamic system. It can represent the optimal properties of the underlying dynamical system only if the embedding parameters are properly selected. In this study, time delay δ is empirically estimated using the average mutual information. And embedding dimensionality m is independently assessed by the false nearest neighbors.

Next, at any time point t, we find $L-1-\left(m-1\right)\delta$ nearest neighbors to $Y_{t}^{m,\delta }$ from the shadow manifold $M_{Y}$, which is labeled $\left\{t_{1},t_{2},\ldots ,t_{L-\left(m-1\right)\delta }\right\}$ from near to far as:

(6) $$\begin{array}{c} M_{Y,t}=\left[Y_{t_{1}}^{m,\delta },Y_{t_{2}}^{m,\delta },\ldots ,Y_{t_{L-1-\left(m-1\right)\delta }}^{m,\delta }\right] \end{array}$$

Herein, *K* nearest neighbor algorithm $\left(K=m+1\right)$ is used to find these neighbor sets $M_{Y,t}$. To improve the quantization stability, we advance CCM by exploiting all the remaining embedding vectors in the attractor manifold. Using all these nearest neighbor points, the estimation of $x\left(t\right)$ are calculated as follows:

(7) $$\begin{array}{c} \hat{x}\left(t\right)=\sum \omega_{k}x\left(t_{k}\right) \end{array}$$

where $\omega_{k}$ is the associated weight at time $t_{k}$, $\omega_{k}=\frac{d_{k}}{\sum d_{k}},k=1,2,\ldots ,L-1$. $t_{k}$ represents the time of the kth nearest neighbor of $Y_{t}^{\prime }$.

(8) $$\begin{array}{c} d_{k}=\exp\left\{-\frac{\left\|y\left(t_{k}\right)-y\left(t\right)\right\|}{\left\|y\left(t_{1}\right)-y\left(t\right)\right\|}\right\} \end{array}$$

where $\left\|y\left(t_{k}\right)-y\left(t\right)\right\|$ is the Euclidean distance between two vectors $y\left(t_{k}\right)$ and $y\left(t\right)$. In this calculation, $\hat{x}\left(t\right)$ is estimated by full nearest neighbors in shadow manifold $\tilde{M_{Y}}$ and raw series $x\left(t\right)$, therefore we call it “full cross mapping”.

Finally, we compute the Pearson's correlation coefficient between the estimated series $\hat{x}\left(t\right)$ and raw series $x\left(t\right)$, which is defined as the FCCM:

(9) $$\begin{array}{c} \text{FCCM}\left(X\to Y\right)=\frac{\sum \left(x\left(t\right)-\bar{x\left(t\right)}\right)\left(\hat{x}\left(t\right)-\bar{\hat{x}\left(t\right)}\right)}{\sqrt{\sum {\left(x\left(t\right)-\bar{x\left(t\right)}\right)}^{2}\sum {\left(\hat{x}\left(t\right)-\bar{\hat{x}\left(t\right)}\right)}^{2}}} \end{array}$$

In this research, to identify causal network features with significant differences between surgical success and failure group in EZ, we first assessed the normality of the data using the Lilliefors test. Since the data did not meet the assumption of normality, we applied a one‐tailed non‐parametric Wilcoxon rank sum test and calculated the effect size as Cohen's *d* score. Results were declared significant for p<0.05. To correct multiple comparisons, we applied Benjamini–Hochberg false discovery rate correction at a significance level of 5%.

### 2.5 Machine Learning Classification

For the surgery outcome prediction, we undertook the classification between surgery success and failure group. First, we extracted a total of 4 × 7 = 28 features, including the mean, variance, maximum, and minimum of FCCM results in seven frequency bands for the EZ regions. Based on the correlation of each feature with surgical outcomes, we integrated the top 10 features with the highest Pearson correlation coefficient into a unified feature matrix. Four classifiers, support vector machine (SVM), random forest (RF), k‐nearest neighbor (KNN) and linear discriminant analysis (LDA) were applied in the prediction. The kernel function for the SVM was specified as a Gaussian kernel and we employed Bayesian optimization to modify the SVM's hyperparameters during cross‐validation. Considering the trade‐off between model performance and computational efficiency, the RF model was configured with 10 sub‐trees and a minimum leaf size of 2 so that the model exhibited consistently good average performance in multiple experiments. For the KNN model, as *K* the number of nearest neighbors, increases, the validation error will exhibit a trend of initially decreasing and then increasing. As a result, we set K=5 corresponding to the minimum point of the validation error as the model parameter. The unified feature matrix was inputted to these four classifiers to train machine learning models for prediction. The performance of these classifiers was quantified using metrics such as accuracy, precision (PPV), NPV, sensitivity, specificity and F1‐score.

## 3 Results

### 3.1 Numerical Experiment Results for Performance Evaluation of FCCM

To verify performance of the developed FCCM, we performed the following numerical experiments and compared with several commonly used causal brain network algorithms. All operation codes ran on a PC with Intel Core i7‐12700F @ 2.1 GHz, 64.0 GB RAM, Windows 10 operating system, and MATLAB R2022b (The MathWorks Inc.). The synthetic dataset used in numerical experiments is a nonlinear coupling dynamics system, modeled as [38, 39, 40]:

(10) $$\left\{\begin{array}{c} \begin{array}{c} x\left(t+1\right)=3.4x\left(t\right)\left(1-x^{2}\left(t\right)\right)e^{-x^{2}\left(t\right)}+\varepsilon_{X}\\ y\left(t+1\right)=3.4y\left(t\right)\left(1-y^{2}\left(t\right)\right)e^{-y^{2}\left(t\right)}+C_{1}y\left(t-\tau_{1}\right)x\left(t\right)+\varepsilon_{Y} \end{array}\\ z\left(t+1\right)=3.4z\left(t\right)\left(1-z^{2}\left(t\right)\right)e^{-z^{2}\left(t\right)}+C_{2}z\left(t-\tau_{2}\right)y\left(t\right)+\varepsilon_{Y} \end{array}\right.$$

where $\varepsilon_{X}$, $\varepsilon_{Y}$, and $\varepsilon_{Z}$ are white Gaussian noise with standard deviation of σ. The numerical model is widely applied in previous studies, and it is an appropriate simulation for evaluating the causality measure accuracy containing nonlinear relationships. There are obviously nonlinear connections: X→Y and Y→Z in Equation (10), and the parameter $C_{1}$ and $C_{2}$ indicate the coupling strength. The system noise ($\varepsilon_{X},\varepsilon_{Y},\varepsilon_{Z})$ in either variable in Equation (10) propagates to later values of the variable and to another variable through coupling terms.

In causal coupling analysis, Granger causality (GC) and transfer entropy (TE) are the two most commonly used methods. Based on these two algorithms, many variants have been proposed to enhance their quantification performance in more complex and multivariable systems. Moreover, CCM was first proposed in 2012 [41] and has been applied in various fields including EEG analysis [42]. As a result, in this paper, the baseline methods used for comparative study include GC, conditional Granger causality (CGC), kernel‐Granger causality (KGC), TE, partial transfer entropy (PTE), symbolic transfer entropy (STE), and CCM, [40, 41, 43, 44, 45, 46, 47, 48, 49, 50]. GC is a straightforward method for assessing causal relationships, while it is limited to linear systems. CGC builds upon GC by incorporating the influence of additional variables, which can enhance the accuracy of causal inference to some extent and performs well in multivariable contexts, yet it remains rooted in linear assumptions. KGC extends the applicability of GC by employing kernel methods to accommodate nonlinear relationships. TE effectively captures the information transfer between variables, being applicable to both linear and nonlinear relationships; nevertheless, it entails high computational complexity and requires a substantial sample size. PTE is advantageous in that it can mitigate the influence of other variables, thus aiding in the control of confounding factors. STE is well‐suited for analyzing discrete data and exhibits robustness to noise, while the process of symbolization may result in the loss of information. Additionally, CCM demonstrates efficacy in capturing causal relationships within nonlinear and dynamic systems, yet it is sensitive to noise.

**FIGURE 2:**
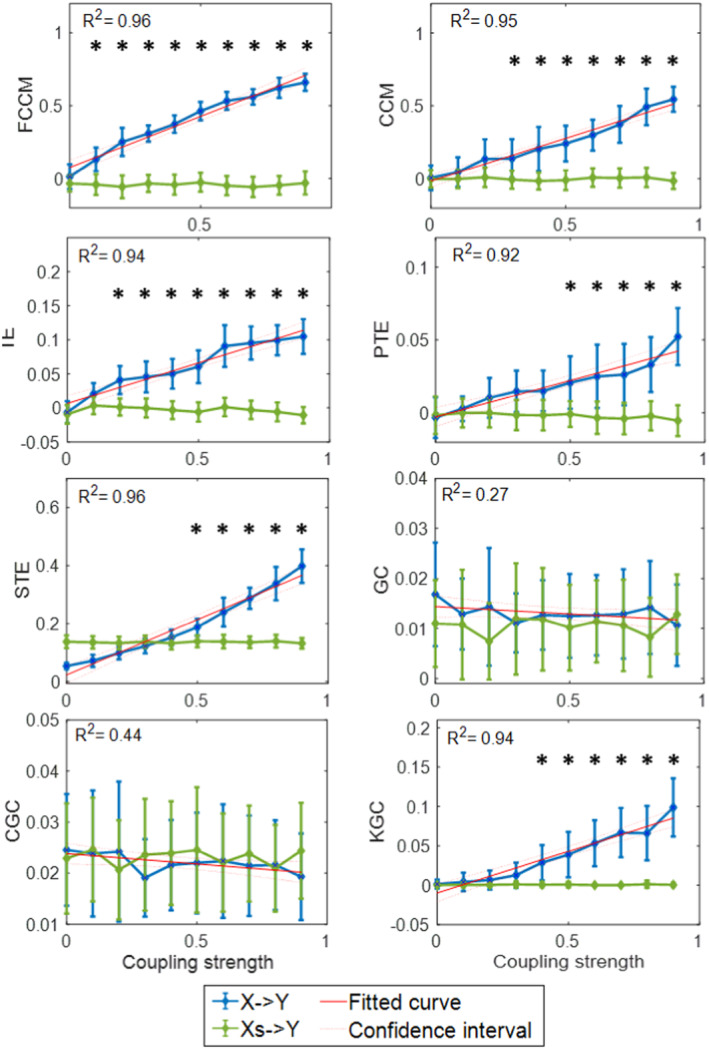
Coupling calculation results (mean ± std) of noisy model X→Y versus coupling strength *C* computed by FCCM and other baseline methods. The red line is fitted to data, and *R* ^2^ is the coefficient of determination of fitting. The shuffled causal coupling Xs→Y is computed after randomly shuffling raw series, and Xs→Y is considered as no causal connection. Marker “*” indicates there is a significant difference between X→Y and Xs→Y; conversely, there is no causal coupling from *X* to *Y*.

By varying the coupling parameters of synthetic model, we first tested the FCCM method's quantification accuracy. The system noise interference σ was set as 0.001, and the coupling parameter $C_{2}$ was set as 0.3. To reduce chance errors and ensure the reliability, we performed 100 realizations in each simulation condition by randomizing the initial values of the synthetic models. When parameter $\;C_{1}$ increased from 0.1 to 0.9 with a step of 0.1, the causality results were calculated by FCCM and other baseline methods. The shuffled causal connectivity Xs−>Y was also computed after randomly shuffling raw series, and Xs−>Y was considered as no causal coupling [51]. Statistically significant differences, marked as “*” in Figure 2 means that the estimation obtains the exact causal coupling from *X* to *Y*.

Theoretically, the causality results will rise with increasing parameter $\;C_{1}$ linearly. The mean value of 100 implementations is sketched versus the parameter $\;C_{1}$ along with its standard deviation as an error bar. The causality results against $C_{1}$ along with the fitted curve are plotted in Figure 2. The coefficients of determination ($R^{2}$) are calculated to check the fitting, which indicates the accuracy of coupling estimation. From the results in Figure 2, the FCCM method can accurately identify causal relationships with an $R^{2}$ of 0.96, which is higher than other methods. This indicates that FCCM method can accurately quantify causal strength.

The coefficients of determination for the CCM and TE methods are $R^{2}=0.95$ and $R^{2}=0.96$, respectively, which also confirmed the good quantification performance of these two methods to some extent. However, when the coupling parameters are low, that is, for the weaker causal relationships, all baseline methods perform the limited quantification accuracy: there is a partial lack of significant differences, meaning they cannot infer the real existing causal coupling X→Y. As for GC and CGC, they are thoroughly invalid in these nonlinear causal systems. In conclusion, the findings in Figure 2 confirm the developed FCCM's highest quantification accuracy.

**FIGURE 3:**
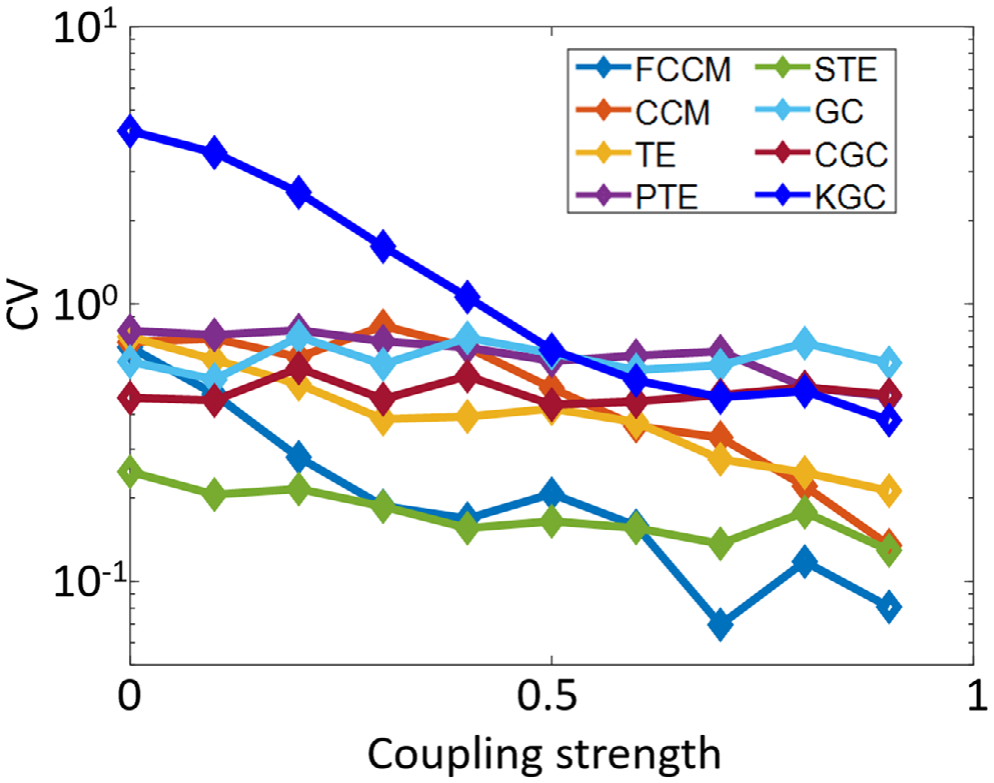
The quantitative stability evaluation results (logarithmic CV values) of the FCCM and other baseline methods in computing X−>Y modeled by Equation (10).

The coefficient of variation (CV) is further introduced to assess quantization stability [52], which is defined as:

(11) $$\mathrm{CV}=\frac{\mathrm{std}\;\left(\left|\text{FCCM}\;\left(X\to Y\right)\right|\right)}{\text{mean}\;\left(\left|\text{FCCM}\;\left(X\to Y\right)\right|\right)}$$

where $\text{mean}\;\left(\text{FCCM}\;\left(X\to Y\right)\right)$ represents the mean value of all FCCM results under the same simulation condition of numerical experiment. Thus, a smaller CV value implies less variability in the multivariate quantization, that is, the algorithm is more stable. The corresponding CV results of causal estimation X−>Y are represented in Figure 3. In general, FCCM and STE have smaller CV values in the range of $C\in \left[0\sim 0.9\right]$, thus further confirming the excellent quantization stability of the developed FCCM.

### 3.2 Statistical Results of Causal Brain Network

During the ictal period of seizures in every frequency band, we employed a sliding window of 10 s without overlaps to extract 1~15 segments for each patient. In total, 205 segments were extracted, including 91 from surgical success group and 114 from surgical failure group. To quantify the causal strength, we calculated the causality through FCCM methods. The correlation coefficients between the 28 extracted features and the surgical outcomes are listed in Table 2.

**TABLE 2:** Correlation coefficients between features and surgical outcomes.

Frequency band	Mean	Variance	Maximum	Minimum
Full (0.5~250 Hz)	−0.1181	**0.5087**	0.0714	−0.3016
δ (0.5~4 Hz)	−0.1945	**0.4395**	0.2398	−0.2661
θ (4~8 Hz)	−0.2844	**0.5991**	0.1546	−0.2958
α (8~13 Hz)	**−0.3206**	**0.7194**	−0.0179	**−0.4401**
β (13~30 Hz)	0.0269	**0.5766**	0.1849	−0.2001
γ (30~80 Hz)	0.0890	**0.4254**	0.2139	−0.0944
HF (> 80 Hz)	0.2170	**0.4126**	**0.3267**	−0.0672

**FIGURE 4:**
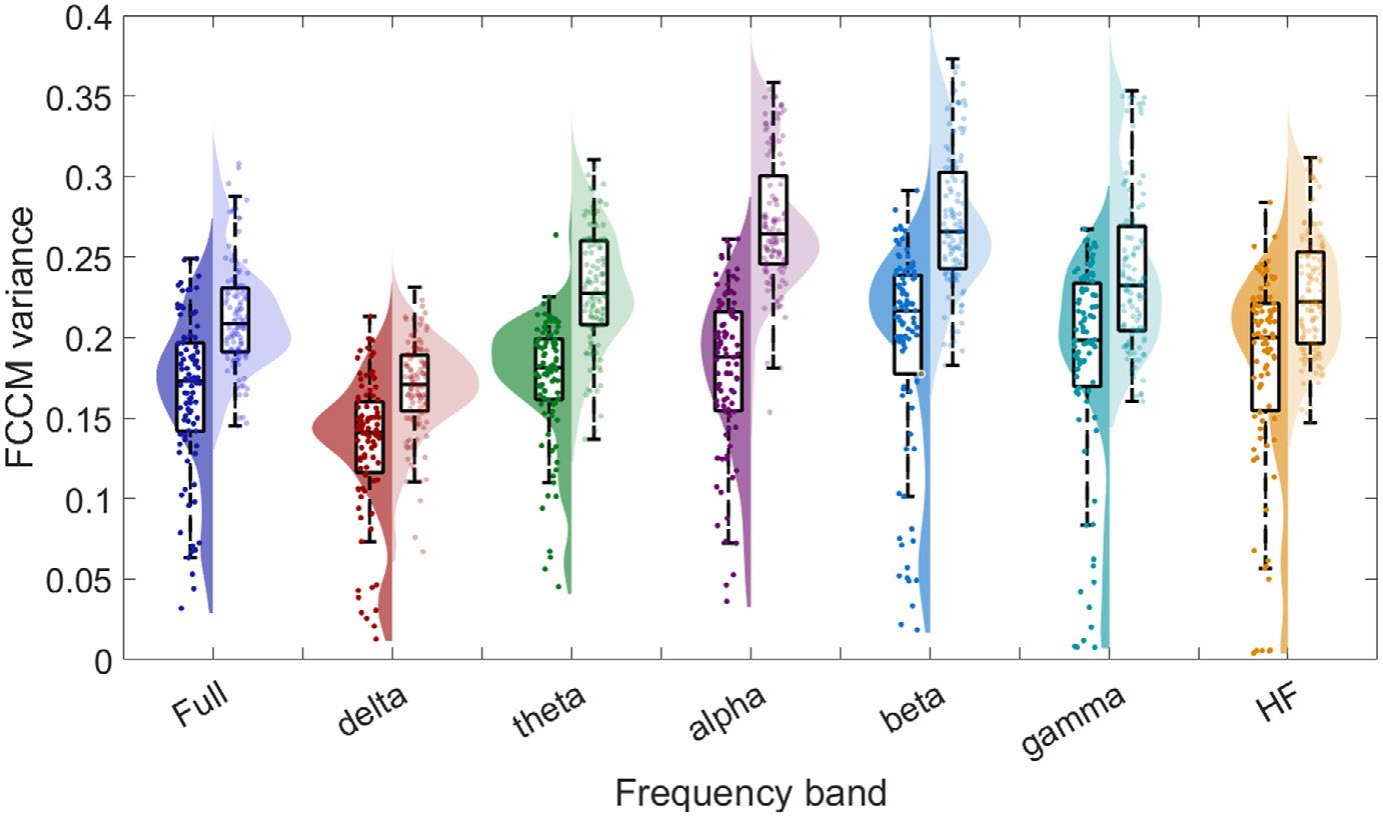
Statistical violin plot of FCCM variance in EZ among 205 segments. For comparison, in each frequency‐band plot, there are surgical success group (the left violin plot filled with saturated color) and surgical failure group (the right violin plot filled with unsaturated color).

In Table 2, the bolded text highlights the top 10 features of epileptic network connectivity with the highest absolute correlation coefficients to surgical outcomes, selected for subsequent surgical outcome prediction using machine learning models. Specifically, Figure 4 presents the statistical results of the variance of FCCM in all frequency. We found that in all frequency bands, the variance of FCCM in EZ of successful surgeries is significantly lower than that of the failed surgeries, indicating that the causal network connectivity between regions within EZ are similar. This might result from the high synchrony in EZ of patients with DRE.

**FIGURE 5:**
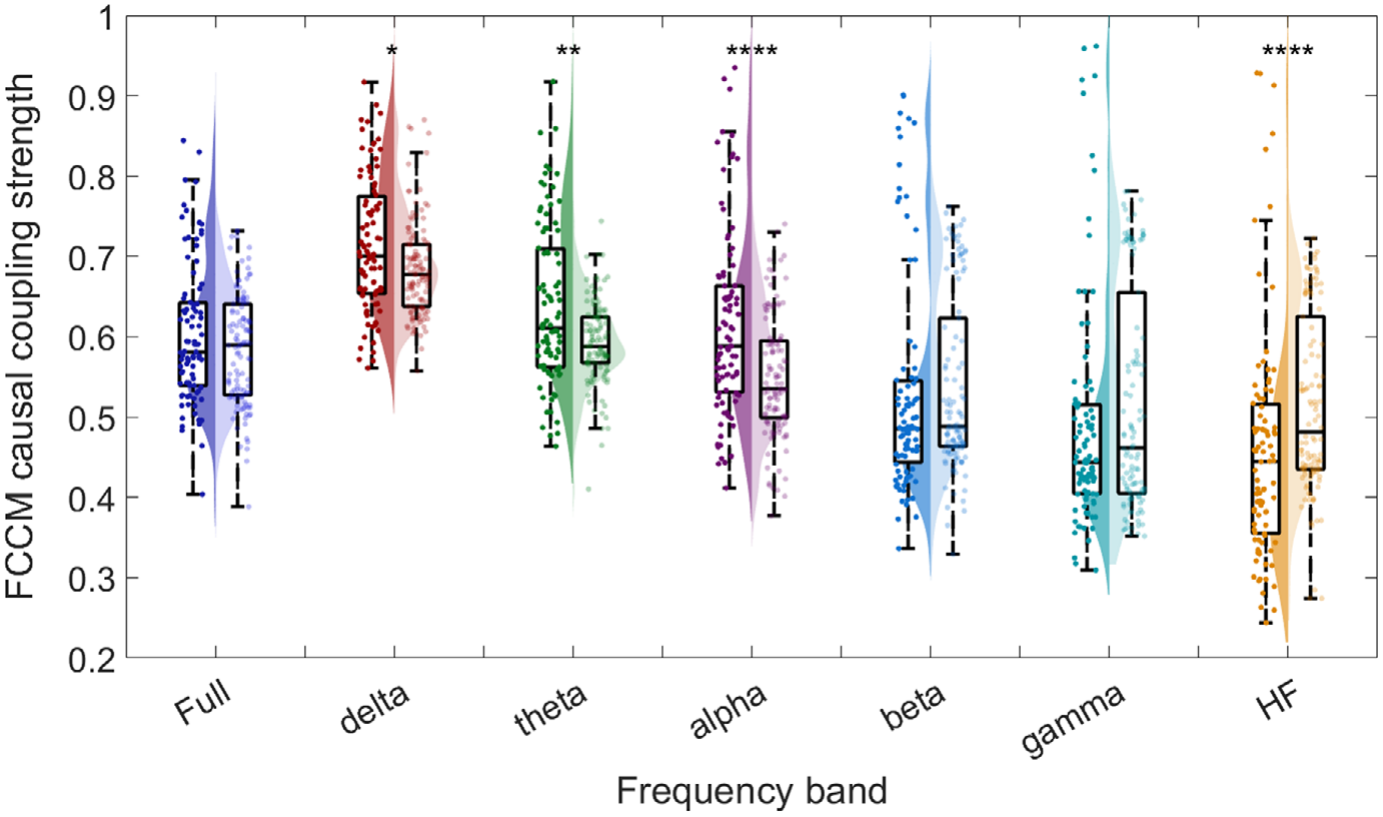
Statistical violin plot of FCCM mean in EZ among 205 segments. In each frequency‐band plot, there are surgical success group (the left violin plot filled with saturated color) and surgical failure group (the right violin plot filled with unsaturated color). *: *p* < 0.05; **p < 0.01; ****p < 0.0001.

We further studied the mean FCCM results in EZ of different frequency bands. The statistical results are shown in Figure 5 and *p*‐value calculated through Wilcoxon rank sum test and effect size are listed in Table 3. We found that in EZ regions, the causal strength of surgical success group and failure group expressed significant differences in *δ*‐, *θ*‐, *α*‐, and HF‐ frequency bands, among which the difference in α‐ frequency band is the most significant with an effect size of 0.68.

**TABLE 3:** Results of statistical analysis among 205 segments.

Frequency band	S vs. F in EZ	
	*p*	Effect size
Full (0.5~250 Hz)	0.2857	0.24
δ (0.5~4 Hz)	**0.0181** (*)	0.40
θ (4~8 Hz)	**0.0046** (**)	0.59
α (8~13 Hz)	**5.00e‐05** (****)	0.68
β (13~30 Hz)	0.3021	−0.05
γ (30~80 Hz)	0.2966	−0.18
HF (> 80 Hz)	**8.58e‐05** (****)	−0.44

*Note:* Bold values mean the best predictive performance in test set. Significance of *: *p* < 0.05; **p < 0.01; ***p < 0.001; ****p < 0.0001.

Abbreviations: F, surgical failure group; S, surgical success group.

**FIGURE 6:**
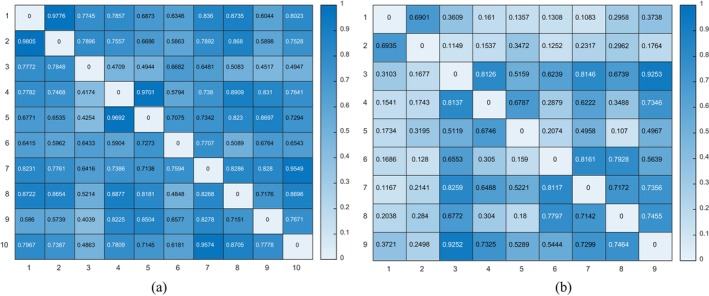
Typical causal strength results in α‐frequency band of patients in surgical success and failure group: (a) surgical success group (S001); (b) surgical failure group (S008).

Here, we exhibited typical α‐frequency band causal strength results of patients in surgical success and failure group. Figure 6 gives an example of S001 in surgical success group and an example of S008 in surgical failure group. The number of rows and columns in Figure 6 correspond to the number of channels in the EZ region. As the channels in the EZ region may vary for each patient, the number of rows and columns may differ accordingly. Through the heatmaps of causal strength, we can intuitively observe that the causal strength in EZ is significantly higher in the surgical success group compared to the surgical failure group, with darker colors indicating higher strength. For patients with DRE, this enhancement suggests abnormal activation in the corresponding brain regions in EZ, which plays a positive role in studying the pathogenesis of epilepsy. The lower causal connectivity strength in certain channels of the surgical failure group suggests that these channels corresponding to the resection zone may not belong to the EZs, which would prompt clinicians to remove these regions or redefine the surgical area to include other potential regions, thereby enhancing the surgical success rate and avoiding the risk resulting from failure.

**FIGURE 7:**
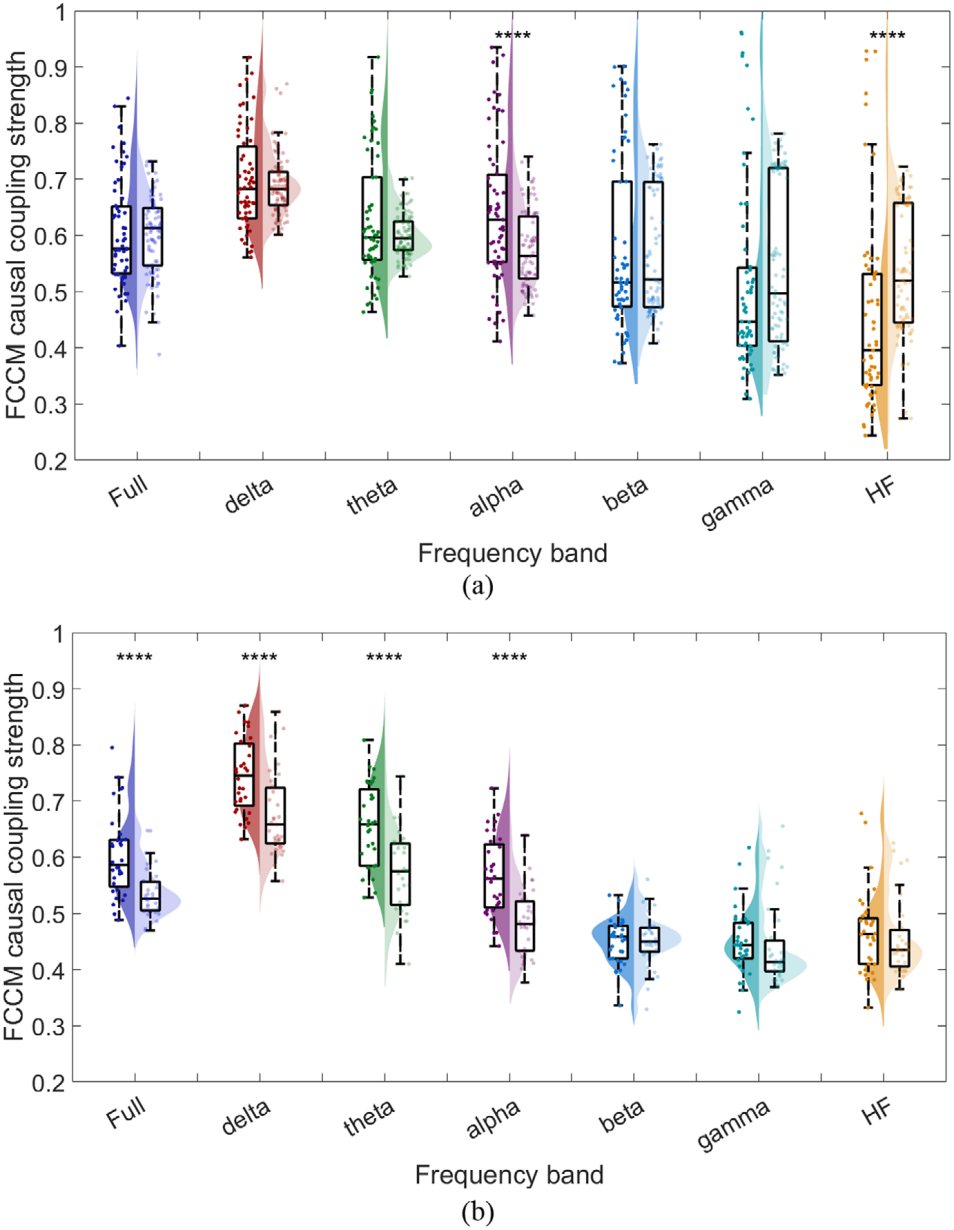
Statistical violin plot of FCCM results: (a) lesional patients, (b) non‐lesional patients. For comparison, in each frequency‐band plot, there are surgical success group (the left violin plot filled with saturated color) and surgical failure group (the right violin plot filled with unsaturated color). ****p < 0.0001.

To further investigate the differences in causal strength among different types of patients, we separately compiled the FCCM results of lesional and non‐lesional patients. The results are shown in Figure 7. And to quantify the differences, Wilcoxon rank sum test was performed and Cohen's *d* effect size was calculated, with results listed in Table 4. According to Figure 7 and Table 4 shown below, we found that there existed differences in causal intensities between lesional and non‐lesional patients. For lesional patients, significant differences exist in α‐ and HF‐ frequency bands, while for non‐lesional patients, significant differences exist in Full‐, *δ*‐, *θ*‐, and *α*‐ frequency bands. But it is worth noting that in both groups, α‐ frequency band exhibited the largest effect size, indicating that across different types of patients, the differences of causal strength in α‐ frequency band are the most meaningful one for further research.

**TABLE 4:** Results of statistical analysis of lesional and non‐lesional patients.

Frequency band	S vs. F in lesional patients		S vs. F in non‐lesional patients	
	*p*	Cohen's *d* effect size	*p*	Cohen's *d* effect size
Full (0.5~250 Hz)	0.4562	0.01	**1.15e‐04** (****)	1.07
δ (0.5~4 Hz)	0.8490	0.11	**2.00e‐04** (****)	0.98
θ (4~8 Hz)	0.6911	0.37	**1.44e‐04** (****)	1.12
α (8~13 Hz)	**7.76e‐04** (****)	0.69	**1.63e‐05** (****)	1.24
β (13~30 Hz)	0.9571	0.04	0.9945	−0.04
γ (30~80 Hz)	0.1439	−0.19	0.1062	0.09
HF (> 80 Hz)	**1.97e‐05** (****)	−0.58	0.3606	0.14

*Note:* Bold values mean the best predictive performance in test set. ****p < 0.0001.

Abbreviations: F, surgical failure group; S, surgical success group.

### 3.3 Surgical Outcome Prediction

To assess the predictive performances of these classifiers, five‐fold cross‐validation was employed. The results are presented in Table 5. The few rows with shaded background display the performance of each model on the training set, while the other rows with white background demonstrate the performance on the validation set. Since the KNN method selects the *K* nearest training samples to help determine the class of a test sample, the metrics for the KNN training set are meaningless and are omitted here. Among these four selected classifiers, the SVM model with Gaussian kernel function and Bayesian optimization exhibits the best performance, achieving an average accuracy of 90.73%, PPV of 87.91%, NPV of 92.98%, sensitivity of 90.91%, specificity of 90.60% and F1‐score of 89.39% as indicated by the bold text in Table 5. The other three models also display satisfying performance with an average accuracy at 90% approximately, indicating the feasibility of the proposed systematic technical route to predict neurosurgical outcomes in patients with DRE. Finally, we compare our work with previous studies on DRE surgical outcome prediction. Both invasive and noninvasive predictions are included. The performance comparisons are listed in Table 6, which further confirms that our work achieves the best prediction. The second‐best prediction results in only 85% accuracy and 87% NPV, and additional performance such as sensitivity, specificity and F1‐score are not reported.

**TABLE 5:** Five‐fold cross‐validation surgical outcome predicting performances.

Classifier	Performance					
	Accuracy (%)	PPV (%)	NPV (%)	Sensitivity (%)	Specificity (%)	F1‐score (%)
SVM	95.85	94.23	97.15	96.35	95.47	95.28
	**90.73**	**87.91**	**92.98**	**90.91**	**90.60**	**89.39**
RF	99.27	99.45	99.12	98.91	99.56	99.18
	90.24	90.11	90.35	88.17	91.96	89.13
KNN	89.76	90.11	89.47	87.23	91.89	88.65
LDA	90.12	92.85	87.94	86.01	93.91	89.30
	88.29	91.21	85.96	83.84	92.45	87.37

*Note:* Bold values mean the best predictive performance in test set.

**TABLE 6:** The comparison of prediction performance between this paper and previous studies.

Ref.	Data recordings	MRI pathology	Feature/Methodology	Accuracy	PPV	NPV	Sensitivity	Specificity	F1‐score
This paper	EEG	N/P	Causal strength in EZ	90.73%	87.91%	92.98%	90.91%	90.60%	89.39%
[6]	iEEG	N/P	Functional connectivity	45%~72%	66%~100%	31%~66%	\	\	
[17]	iEEG/MRI	N	Structural/functional brain networks	85%	\	87%	\	\	
[18]	foramen ovale EEG	N/P	Power spectral densities/logistic regression	68%	\	57%	\	\	\
[7]	iEEG	N/P	Spike propagation in source space	69%	79%	56%	\	\	
[21]	SEEG	P	High‐frequency oscillations	69.5%	60.7%	74.7%	58.6%	76.3%	
[22]	SEEG	P/N	SEEG desynchronization	\	\	\	89.3%	82.4%	\
[24]	EEG/MEG	N/P	Functional connectivity	66%~76%	65%~81%	63%~76%	\	\	
[53]	PET/MRI	N	Feature of hypometabolism	70%~80%	83%~87%	53%~72%	69%~86%	61%~73%	
[54]	PET/FDG	N	Individual metabolic connectome	83.59%	\	75.00%	92.79%	\	
[55]	iEEG	N/P	Neural fragility, structured RF	76%	90.3%	87.2%	\	\	
[32]	EEG/MRI	N/P	Ripple onset	71%	91%	59%			
[56]	EEG/MRI	P/N	Logistic regression	72%	\	\	\	\	

Finally, we compare our work with previous studies on the prediction of DRE surgical outcomes. Both invasive and noninvasive prediction are included. The performance comparisons are listed in Table 6, which further confirms that our work has achieved the best prediction.

## 4 Discussion

In this work, we comprehensively analyzed causal brain networks in all frequency bands, utilizing FCCM method to calculate the causal intensities of epileptogenic brain networks. We found that in some frequency bands, especially in α‐band, the mean causal intensities in EZs of the surgical success group are significantly higher than those of the surgical failure group and the variance is significantly lower in the success group, indicating abnormal activation and high synchrony in the EZ region of DRE patients. For previous studies, it was found that slower alpha rhythm and shifted spatial topography are associated with the poor seizure‐control [57]. Moreover, coexistence of increased functional connectivity in low‐alpha frequency band and decreased functional connectivity in high‐alpha band among cortical regions reflects the complicated physiological interdependence within alpha bands in epilepsy [58]. The results of ours are consistent with the conclusions of these previous studies.

**FIGURE 8:**
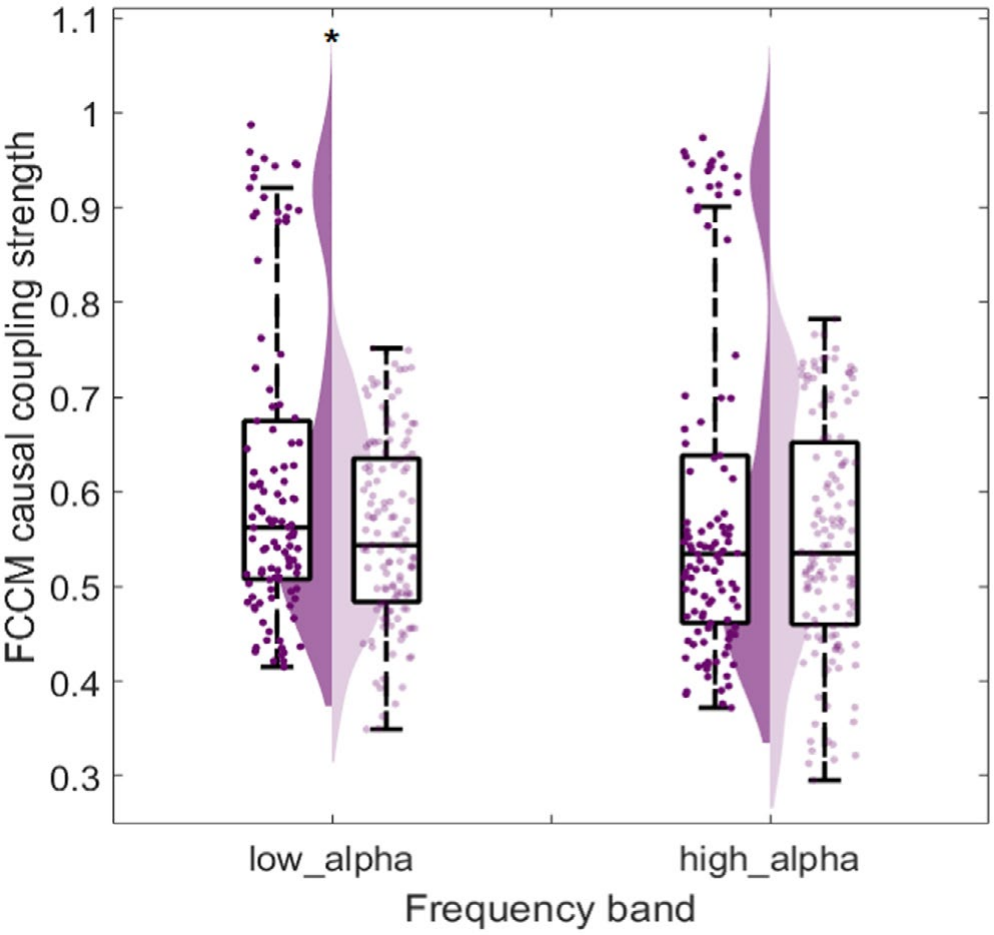
Statistical violin plot of FCCM mean in EZ. For comparison, in each frequency‐band plot, there are surgical success group (the left violin plot filled with high saturation color) and surgical failure group (the right violin plot filled with low saturation color). *p < 0.05.

Furthermore, we consequently explored the causal coupling strength in low‐alpha (8~10 Hz) and high‐alpha (10~13 Hz) frequency bands. The *p*‐values of Wicoxon ranksum test and Cohen's *d* score are listed in Table 7 and the violin plot are shown in Figure 8. We found that in low‐alpha frequency band, there existed significant difference in the mean of FCCM results between surgical success and failure groups, with a Cohen's *d* effect size of 0.4365. This finding demonstrates that the causal connectivity within EZ would increase during seizures in low‐alpha band, which aligns with the result in [58]. Moreover, Based on the results in Table 7 and Figure 8, when the α band was further subdivided into low‐alpha and high‐alpha bands for experiment, the significance in the low‐alpha and high‐alpha bands was not as pronounced as in α band. Therefore, directly using the α band, rather than a more refined band division, is more appropriate, as finer band divisions might not be necessary for the analysis in this experiment.

**TABLE 7:** Results of statistical analysis in low‐ and high‐ α frequency bands.

Frequency band	*p*	Cohen's *d*
Low‐ α (8~10 Hz)	**0.0201**	**0.4365**
High‐ α (10~13 Hz)	0.7358	0.2470

*Note:* Bold values mean the best predictive performance in test set.

Considering different DRE types, i.e., lesional and non‐lesional DRE, source causal brain networks were further statistically analyzed separately. FCCM‐defined EZ causal connectivity seems more significant differences between successful and failed surgeries for non‐lesional patients with DRE, with p<0.0001 in Full, δ,θ,andα‐band networks; while, p<0.0001 in α and HF‐band networks in distinguishing lesional DRE's surgical outcomes. The lesional DRE's brain network generate a heterogeneous complex system. When FCCM quantifies the causal coupling in complex system, it is based on the non‐separability of system variables, and more attention is paid to the system's homogeneity. Subsequently, we performed machine learning prediction achieving a satisfying average accuracy at 90.73%, higher than the previous studies. This can be attributed to the success of the FCCM method in capturing dynamic information related to network changes within the EZ during EEG ictal segments. Based on our predictive results, clinicians can ascertain whether the delineated surgical areas truly belong to the EZs, allowing them to refine the surgical or intracranial electrodes implantation plans, so that the treatment outcomes will be improved and the risk of surgical failure will be mitigated. Additionally, the method proposed in this paper can also be utilized for the auxiliary diagnosis of other neurological disorders. For instance, in the brains of patients with autism or Alzheimer's disease, there might be abnormal functional connectivity. The brain network analysis method presented in this paper, FCCM, can capture these atypical connectivity patterns, providing insights into the pathophysiological mechanisms of these diseases.

It should be noted that the present study still has several limitations. We used the default anatomical and standard electrode positions when performing the EEG source imaging analysis. For further investigation, the individual MRI data corresponding to each patient could be added in order to perform a more accurate source mapping. More importantly, the statistical results of lesional and non‐lesional patients demonstrate that there are some differences among different types of DRE patients. As a result, subsequent steps could involve employing transfer learning methods to achieve model generalization across different patient types, or constructing a multi‐task model to initially classify and predict the input patient types, and then predict surgical outcomes. As a prospective single‐center study, the dataset, especially the data of non‐lesional patients, remains limited. Therefore, future efforts may involve supplementing the dataset with multi‐center, multi‐type patients to train a model that better aligns with the overall distribution of causal strength in the EZ. Despite these limitations, our study contributes significantly to the noninvasive diagnosis and treatment of DRE by proposing a viable framework for predicting neurosurgical outcomes.

In conclusion, for noninvasively predicting neurosurgical outcomes in patients with DRE, we have proposed a systematic framework, source causal connectivity. Moreover, we applied a robust causal network measuring method, FCCM, to compute the causal strength in the EZ. Based on the Wilcoxon rank sum test and Cohen's *d* effect size, we found that in α frequency band, the difference between surgical success and failure group is the most significant with the lowest P‐value at 5.00e‐05 and the largest effect size at 0.68, indicating that the causal network features are closely correlated with DRE surgical outcomes. Finally, we used four kinds of machine learning classifiers to predict neurosurgical outcomes based on the features extracted from causal intensities. The results showed that we achieved the state of the art (SOTA) performance on surgical outcome prediction for patients with DRE.

## Conflicts of Interest

The authors declare no conflicts of interest.

## Data Availability

The source code and sample data supporting this study are publicly available on GitHub: https://github.com/wyl1994/Source‐code‐and‐supporting‐dataset‐for‐CNS‐Neuroscience‐Therapeutics‐paper/tree/main. The source data are not publicly available due to privacy or ethical restrictions.
